# CycloZ Suppresses TLR4-Driven Inflammation to Reduce Asthma-Like Responses in HDM-Exposed Mouse Models

**DOI:** 10.3390/cells13232034

**Published:** 2024-12-09

**Authors:** Dohyun Lee, Jongsu Jeon, Seoyeong Baek, Onyu Park, Ah-Ram Kim, Myoung-Sool Do, Hoe-Yune Jung

**Affiliations:** 1 R&D Center, NovMetaPharma Co., Ltd., Pohang 37668, Republic of Korea; 2 School of Life Science, Handong Global University, Pohang 37554, Republic of Korea; 3 Department of Advanced Convergence, Handong Global University, Pohang 37554, Republic of Korea; 4 School of Interdisciplinary Bioscience and Bioengineering, Pohang University of Science and Technology (POSTECH), Pohang 37673, Republic of Korea

**Keywords:** asthma, HDM, inflammation, TLR4

## Abstract

Asthma is a chronic lung disease characterized by airway inflammation, hyperresponsiveness, and narrowing, with a risk of life-threatening attacks. Most current treatments primarily consist of inhalable steroids, which are not without adverse effects. Recently, there has been growing interest in alternative approaches to asthma management. In this study, we investigated the anti-asthmatic effects of the non-steroidal compound CycloZ using acute and chronic mouse models of asthma. Allergic reactions were induced with house dust mite (HDM) extract, and CycloZ or fluticasone propionate (FP) was administered orally or intranasally, respectively. CycloZ significantly ameliorated the HDM-induced robust expression of Th2 cytokines in both models. CycloZ also decreased immune cell infiltration into the lungs and reduced IL-4 and IL-13 cytokine levels in bronchoalveolar lavage fluid (BALF). Moreover, CycloZ greatly attenuated the activation of the TLR-4 pathway, which is involved in HDM recognition and signaling. The beneficial effects of CycloZ were comparable to or even superior to the current steroid treatment, FP, suggesting that CycloZ could be a promising new option for asthma therapy.

## 1. Introduction

Asthma, a chronic lung disease with narrowed and swollen airways and excessive mucus production, leads to difficulty in breathing [1,2]. Asthma is a minor nuisance for some people, but for others, it can be a major problem that interferes with daily activities and leads to a life-threatening asthma attack [2]. A total of 339 million people worldwide were affected by asthma in 2023, and the prevalence is still increasing [3]. The causes of asthma are complex as both genetic and environmental risk factors such as house dust mites (HDMs), pollen, mold spores, or cockroach waste can contribute to the development of asthma [4].

HDMs are a major allergen contributing to the development and exacerbation of asthma, inducing allergic responses in 85% of asthmatic individuals [5,6]. HDMs’ exogenous proteases, Der p1 and Der f1, directly stimulate the respiratory tract and trigger pro-inflammatory responses [5]. These proteases activate toll-like receptors (TLRs) such as TLR4 in lung epithelial cells to activate an important signaling pathway through NF-kB, MAPK, and IRF3 [7,8]. Their activation promotes the release of Th2-type cytokines, such as interleukin-4 (IL-4), IL-5, and IL-13 [8]. IL-4 stimulates B cells to produce IgE antibodies, upregulates IgE receptors on mast cells, and promotes Th2 cell differentiation, thereby enhancing allergic responses and driving the production of IL-5 and IL-13 [9]. IL-5 plays a crucial role in the production, maturation, and activation of eosinophils [10]. IL-13 is important to goblet cell differentiation and mucus production [11]. The upregulation of these Th2 cytokines orchestrates immune cell recruitment and airway hyperresponsiveness, leading to allergic asthma [12].

Currently, several drugs are available for asthma treatment, but most are primarily inhalable steroids and may not be effective for all patients without side effects [13]. Therefore, interest in a new approach to asthma treatment has recently increased [14], and the development of a new drug is required.

CycloZ is composed of cyclo-His-Pro (CHP) and zinc. CHP has been reported to have protective effects against oxidative stress, inflammation, and fibrosis [15,16,17,18]. It also enhances zinc absorption in the intestine [19]. Given the fact that zinc deficiency is observed in asthmatic patients [20,21], CycloZ may help regulate zinc levels. Moreover, the anti-inflammatory activity of CycloZ [22] may reduce allergic responses, and its inhibitory activity on excessive extracellular matrix (ECM) production [18] may prevent airway remodeling in asthma. Based on these hypotheses, our preliminary comparison study revealed that CycloZ was more effective than either CHP or zinc alone in reducing asthmatic cytokine expression (data now shown).

According to the fact that 50–85% of asthmatics worldwide are typically allergic to HDMs and have elevated levels of HDM-specific IgE, HDMs are a clinically relevant allergen for the experimental setting [23]. Therefore, we investigated the therapeutic potential and molecular mechanisms of CycloZ in acute and chronic mouse models of HDM-induced allergic asthma. We also compared the therapeutic efficacy of CycloZ with that of FDA-approved fluticasone propionate (FP) as a reference drug.

## 2. Materials and Method

### 2.1. Animals

6-week-old male and female Balb/c mice for the acute and chronic HDM-induced asthma model and 7-week-old male C57BL/6J mice for the acute ovalbumin (OVA)-induced asthma model were purchased from Hana-biotech (Pyeongtaek, Korea). Mice were housed in group cages at a temperature of 23 ± 3 °C with a 12 h light/dark cycle. Mice were free to access distilled water and a laboratory chow diet ad libitum. Animal experiments were initiated after a week of adaptation. All animal experiments were approved in accordance with the Ethics Review Committee of the Pohang Advanced Bio Convergence Center, Republic of Korea (Approval number: ABCC2022001, ABCC2022101).

### 2.2. Allergen Instillation and Drug Administration

For the acute HDM model, 50 female Balb/c mice were randomly separated into four groups (CTRL, n = 8; HDM + vehicle, n = 14; HDM + CycloZ, n = 14; HDM + FP, n = 14) and mice were sensitized with 25 μg of HDM extract (Greer Laboratories XPB82D3A25 (Lenoir, NC, USA), D. *Pteronyssinus*, 0.28 mg protein/mg dry wt) via intranasal instillation (50 μL volume) on day 0 and day 3. One week later, mice were challenged with 25 μg of HDM extract via intranasal instillation for three consecutive days. The control group (CTRL) was given normal saline. Drugs (CycloZ, 15 mg/kg p.o.; FP, 20 μg/head i.n.) were administered immediately after HDM challenges.

For the chronic HDM model, 22 mice were randomly separated into four groups (CTRL, n = 4; HDM + vehicle, n = 6; HDM + CycloZ, n = 6; HDM + FP, n = 6) and given 50 μg of HDM extract intranasally 3 times per week. At week 4, 15 mg/kg of CycloZ or 50 μg/head of FP started to be administrated 6 times per week for 3.5 weeks.

For the HDM dose response study, 22 each of male and female Balb/c mice were randomly separated into four groups according to sex (CTRL, n = 4; 10 μg HDM, n = 6; 25 μg HDM, n = 6; 50 μg HDM, n = 6). The mice were sensitized with 10, 25, or 50 μg of HDM extract via intranasal instillation (a 50 μL volume) on day 0 and day 3. One week later, mice were challenged with 10, 25, or 50 μg of HDM extract via intranasal instillation for three consecutive days. The control group was given normal saline.

For the acute OVA model, 16 male C57BL/6J mice were randomly separated into three groups (CTRL, n = 4; OVA + vehicle, n = 6; OVA + CycloZ, n = 6), and mice were sensitized with 10 μg of OVA (Sigma-Aldrich, A5503, Saint Louis, MO, USA) and 1 mg of aluminum hydroxide (Sigma-Aldrich, 239186) mixture via intraperitoneal injection (a 100 μL volume) on day 0 and day 7. One week later, mice were challenged with 50μg of OVA via intranasal instillation (a 50 μL volume) for three consecutive days. The control group was given normal saline. A dose of 15 mg/kg of CycloZ was orally administered immediately after OVA challenges.

### 2.3. BALF Collection, and Total Cell Count in BALF

Mice were euthanized 48 h after the last HDM instillation, and a 24-gauge catheter was introduced into the trachea of the mice. BALF was then collected using a 0.5 mL PBS-filled syringe after three gentle injections and aspirations. A volume of 10 μL of BALF was used to count total cells using the EVE™ Automated Cell Counter (NanoEntek, Seoul, Republic of Korea). After cell counting, the BALF was centrifuged at 3000× *g* for 5 min to remove cells and debris. The supernatant was then frozen and stored at −80 °C for subsequent ELISA analysis.

### 2.4. Lung Collection

Following BALF collection, a cardiac perfusion with 10 ml of PBS was performed to remove residual blood in the lungs. After lung dissection, the left lung lobe was fixed in 10% neutral-buffered formalin (Sigma-Aldrich, HT501128) for histological analysis, and the remaining lobes were frozen on dry ice and stored at −80 °C for molecular analysis.

### 2.5. RNA Analysis

Total RNA was extracted from the lung tissues using NucleoZOL (Macherey-Nagel, Düren, Germany, MN740404.200). A total of 1 μg of total RNA was used for cDNA synthesis using the iScript cDNA synthesis kit (Bio-Rad, Richmond, CA, USA, BR1708891). Real-time qPCR was conducted Using IQ IQ SYBR^®^ Green Supermix (Bio-Rad, BR1708884). The protocol included an initial denaturation step at 95 °C for 3 min followed by 40 cycles of denaturation at 95 °C for 10 s, annealing at 60 °C for 10 s, and extension at 72 °C for 30 s. Primer pairs were designed using the Primer-BLAST tool provided by NCBI and the specificity was confirmed through melt curve analysis conducted by gradually heating samples from 65 °C to 95 °C in 0.5 °C increments, holding at each step for 10 s while monitoring fluorescence. The primer sequences are provided in Table S1.

### 2.6. ELISA

The concentrations of IL-4 and IL-13 in the BALF were measured using ELISA kits (Biolegend, San Diego, CA, USA, 431104, and Invitrogen, Carlsbad, CA, USA, 88-7137-88), according to the manufacturer’s instructions. Frozen BALF was thawed on ice and analyzed without dilution. The absorbance at 450 nm was measured for each ELISA plate, and the concentrations were calculated using a standard curve.

### 2.7. Histology

Formalin-fixed lung tissues were embedded with paraffin, sectioned at a thickness of 4 μm, and deparaffinized. The sections were then rehydrated and stained with hematoxylin and eosin (H&E), or periodic acid–Schiff (PAS) stains. The stained sections were visualized using microscopy (Olympus BX53 upright microscope, Tokyo, Japan).

### 2.8. Western Blot

Lung tissues were lysed using an RIPA buffer (Thermo, Waltham, MA, USA, 89901) supplemented with Halt™ Protease and Phosphatase Inhibitor Cocktail (Thermo, Waltham, MA, USA, 78438). Protein lysates (20μg) were separated on Bolt 4–12% Bis-Tris Plus gels (Invitrogen, Carlsbad, CA, USA, NW04125BOX) and transferred to a nitrocellulose membrane. After 1 h of a blocking step with 5% skim milk, the membranes were incubated with primary antibodies against phospho-p38 (CST, Danvers, MA, USA, #9215), p38 (CST, Danvers, MA, USA, #9212), or Gapdh (CST, Danvers, MA, USA, #5174) at 4 °C overnight. Then, the membranes were labeled with a horse radish peroxidase-conjugated secondary antibody (Promega, Madison, WI, USA, W4011) at room temperature for 1 h after three wash steps. With pico-grade ECL solution (DonginLS, Seoul, Republic of Korea, ECL-PS100), chemiluminescent images were captured using Alliance 4.7 (UVITEC, Cambridge, UK). 

### 2.9. Statistics

The statistical analysis of all data was processed using GraphPad Prism v.6.0. All data were expressed as the mean ± the standard error of the mean (SEM). Significant outliers identified using Grubb’s test were excluded. The significance of differences between two groups were analyzed using Student’s *t*-test (two-tailed). A *p*-value less than 0.05 was considered significant.

## 3. Results

### 3.1. CycloZ Alleviates Allergic Asthma Responses in Acute Asthma Models

**Figure 1:**
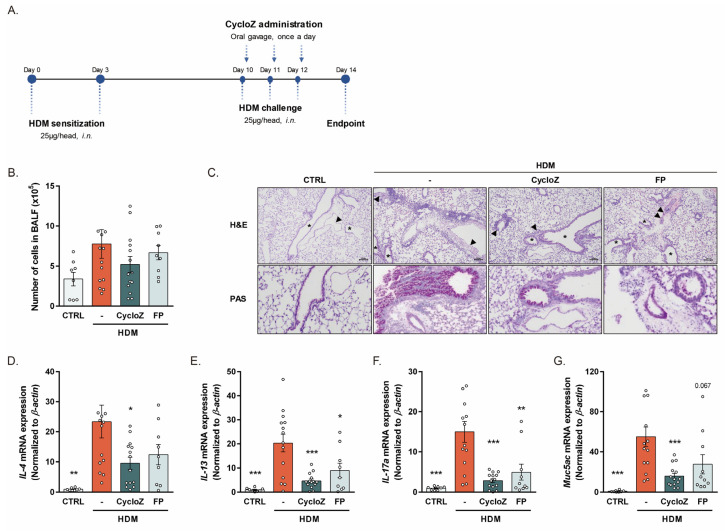
CycloZ reduced allergic asthma responses in HDM-induced acute asthma model mice. (**A**) Experimental design for HDM-induced acute asthma. (**B**) Number of cells in BALF. (**C**) Alveolar macrophage aggregation, chronic inflammation, hypertrophy in all internal and medial pulmonary vessels (arrows), and mucous cell metaplasia in bronchus (stars), shown by H&E stain. Mucinous secretion in bronchus and thickened pulmonary vessel wall shown by PAS stain. Scale bar of 100 μm. (**D**,**E**) Th2-type cytokine *IL-4* and *IL-13* expression in the lung. (**F**,**G**) *IL-17a* and *Muc5ac* expression in the lung. Data are shown as mean ± SEM. Unpaired Student’s *t*-tests. * *p* < 0.05, ** *p* < 0.01, and *** *p* < 0.001 compared with HDM vehicle group.

To establish the HDM-induced acute asthma model, we first exposed male and female mice to three different concentrations of HDM (10 μg, 25 μg, and 50 μg). The significant production of Th2 cytokines (IL-4, IL-13) was observed with doses of 25 μg and 50 μg, with female mice showing a more dramatic response than male mice (Figure S1). Based on these findings, we selected female mice challenged with 25 μg of HDM to investigate the therapeutic potential of CycloZ in acute allergic asthma. To determine the effectiveness of a therapeutic intervention rather than prevention, CycloZ was administered immediately after HDM challenges (Figure 1A). The HDM challenge significantly increased total cell counts in the bronchoalveolar lavage fluid (BALF), a hallmark of lung inflammation. CycloZ treatment showed a tendency to decrease the total cell count, indicating its potential anti-inflammatory effect (Figure 1B). Histological examination revealed marked alveolar macrophage aggregation, acute inflammation, pulmonary vascular hypertrophy, and mucous cell metaplasia in HDM-challenged mice. CycloZ treatment significantly reduced these pathological features, as confirmed by hematoxylin and eosin (H&E) and periodic acid–Schiff (PAS) staining (Figure 1C). At the molecular level, the HDM challenge elevated the expression of major Th2 cytokines (IL-4 and IL-13), IL-17a, and muc5ac, which are key mediators of airway hyperresponsiveness and mucus overproduction. CycloZ treatment significantly downregulated these molecules compared to vehicle treatment, and its effects were superior to those of FP, a commonly used corticosteroid (Figure 1D–G). To further validate the anti-asthmatic potential of CycloZ, we examined its effects in male mice exposed to 25 μg HDM. While male mice exhibited less severe responses compared to females, CycloZ treatment effectively alleviated HDM-induced inflammation and mucus overproduction in this model as well (Figure S2). 

### 3.2. CycloZ Dramatically Ameliorates Asthma Phenotypes in Chronic Asthma Model

**Figure 2:**
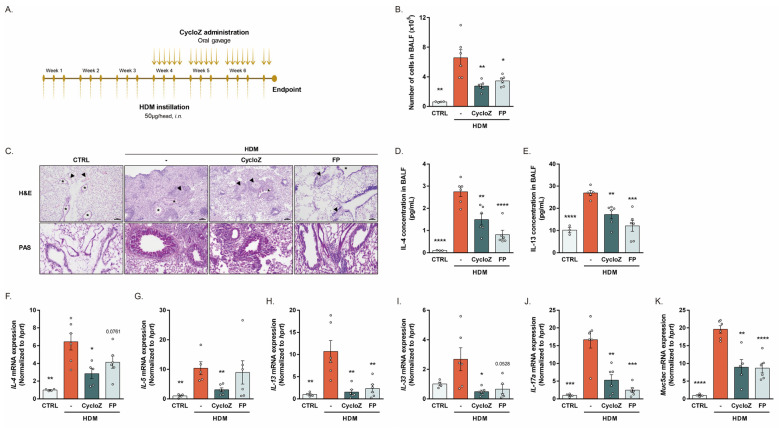
CycloZ recovered asthma phenotypes in HDM-induced chronic asthma model mice. (**A**) Experimental design for HDM-induced chronic asthma. (**B**) Number of cells in BALF. (**C**) Alveolar macrophage aggregation, chronic inflammation, hypertrophy in all internal and medial pulmonary vessels (arrows), and mucous cell metaplasia in bronchus (stars), shown by H&E stain. Mucinous secretion in bronchus and thickened pulmonary vessel wall shown by PAS stain. Scale bar of 100 μm. (**D**,**E**) Levels of IL-4 and IL-13 in BALF. (**F**–**H**) Th2-type cytokine *IL-4*, *IL-5*, and *IL-13* expression in the lung. (**I**–**K**) *IL-33*, *IL-17a*, and *Muc5ac* expression in the lung. Data are shown as mean ± SEM. Unpaired Student’s *t*-tests. * *p* < 0.05, ** *p* < 0.01, *** *p* < 0.001, and **** *p* < 0.0001 compared with HDM vehicle group.

In order to investigate whether CycloZ is also effective for chronic asthma, we employed a prolonged HDM exposure model. Mice received HDM three times per week and for therapeutic intervention, CycloZ or FP was administered after 3 weeks of repeated HDM instillation (Figure 2A). The HDM challenge resulted in a robust increase in total cell counts in the BALF, indicative of chronic airway inflammation. CycloZ treatment significantly reduced the total cell counts compared to the model group, demonstrating its effectiveness in attenuating chronic inflammatory responses (Figure 2B). Histological analyses showed pronounced alveolar macrophage aggregation, chronic inflammation, and mucous cell metaplasia in HDM-challenged mice. CycloZ treatment substantially alleviated these pathological changes, as evidenced by H&E and PAS staining (Figure 2C). Moreover, the HDM-induced elevation of Th2 cytokines, including IL-4 and IL-13, was significantly reduced in the BALF of CycloZ-treated mice (Figure 2D,E). Consistently, the gene expression of Th2-type cytokines such as *IL-4*, *IL-5*, and *IL-13* was significantly reduced by CycloZ, which was more effective than FP treatment (Figure 2F–H). Interestingly, CycloZ significantly reduced the expression of IL-33, a cytokine that promotes Th2 cytokine production and is critical in chronic asthma pathogenesis (Figure 2I). CycloZ also significantly reduced the expression of *IL-17a* and *muc5ac*, further supporting its dual anti-inflammatory and mucus-reducing effects (Figure 2J,K).

### 3.3. CycloZ Reduced the HDM-Induced Upregulation of Th2 Response Modulators

**Figure 3:**
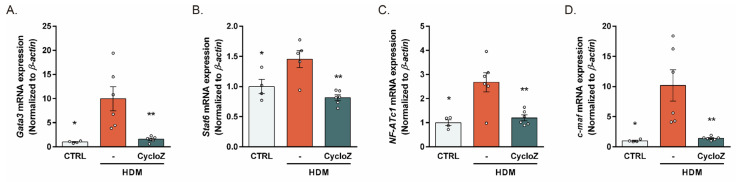
The master regulators of the Th2 response, gata3 and stat6, were downregulated by CycloZ. (**A**,**B**) *Gata3* and *Stat6* expression in the lung. (**C**,**D**) *NF-ATc1* and *c-maf* expression in the lung. Data are shown as mean ± SEM. Unpaired Student’s *t*-tests. * *p* < 0.05 and ** *p* < 0.01 compared with HDM vehicle group.

To further elucidate the mechanism underlying the Th2 response-reducing effects of CycloZ, we investigated the expression of key Th2 response modulators in the chronic model. CycloZ significantly downregulated the expression of *Gata3*, a master regulator of Th2 differentiation [24], as well as its upstream *Stat6* (Figure 3A,B). Additionally, CycloZ significantly reduced the expression of *NF-ATc1* and *c-maf*, downstream targets of *Gata3* that induce Th2 gene transcription (Figure 3C,D). These results indicate that CycloZ effectively inhibits the IL-4-Stat6-Gata3 axis, thereby suppressing the molecular drivers of Th2 inflammation.

### 3.4. CycloZ Inhibited HDM-Driven TLR4 Pathway Activation

**Figure 4:**
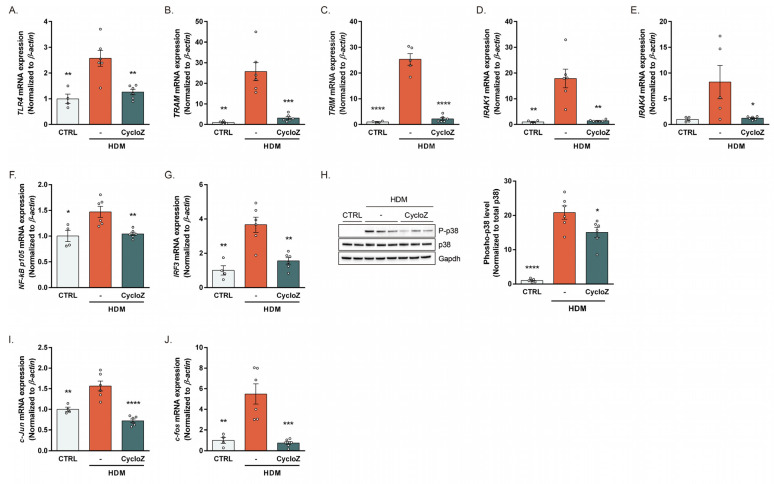
CycloZ also suppressed HDM-induced TLR4 pathway activation. (**A**–**G**) TLR4 pathway gene expression in the lung. (**A**) *TLR4*. (**B**) *TRAM*. (**C**) *TRIM*. (**D**) *IRAK1*. (**E**) *IRAK4*. (**F**) *NF-κB*. (**G**) *IRF3*. (**H**) Representative Western blot image of p38 activation and the quantified blot densities. (**I**,**J**) AP-1 (*c-Jun* and *c-fos*) expression in the lung. Data are shown as mean ± SEM. Unpaired Student’s *t*-tests. * *p* < 0.05, ** *p* < 0.01, *** *p* < 0.001, and **** *p* < 0.0001 compared with HDM vehicle group.

We then investigated whether CycloZ modulates the TLR4 pathway, a key pathway involved in HDM recognition and subsequent Th2 response induction. The HDM-induced TLR4 upregulation was significantly downregulated by CycloZ treatment (Figure 4A). CycloZ also dramatically downregulated the downstream modulators of TLR4, including *TRAM*, *TRIM*, *IRAK1*, and *IRAK4* (Figure 4B–E). Moreover, the downregulation of *NF-κB p105* and *IRF3* by CycloZ (Figure 4F,G) led to an inhibition of p38 MAPK and the expression of AP-1 transcription factors (Figure 4H–J). These results highlight CycloZ’s ability to attenuate HDM-induced TLR4 signaling, potentially dampening the initial immune triggers of allergic inflammation and its downstream effects.

## 4. Discussion

In this study, we evaluated the effects of CycloZ as a therapeutic intervention in acute and chronic mouse models of allergic asthma. HDM exposure led to airway allergic responses such as increased inflammatory cell accumulation in the lungs and expression levels of Th2-type cytokines and several regulators involved in Th2 responses. CycloZ markedly reduced these allergic responses and significantly downregulated the TLR4 pathway, suggesting dual action in reducing inflammation and suppressing the initial immune triggers involved in allergic asthma.

CycloZ effectively lowered the levels of Th2 cytokines (IL-4, IL-13) and related genes in both acute and chronic asthma models, pointing to its potent anti-inflammatory effects. The observed reduction in Gata3 expression underscores CycloZ’s impact on the IL-4-Stat6-Gata3 axis, which plays a crucial role in asthma pathogenesis. However, we also speculated that CycloZ may also exert effects upstream in the allergic cascade, possibly by modifying antigen recognition or signaling, which could amplify its impact on subsequent allergic responses.

Our study also highlights CycloZ’s suppression of the TLR4 pathway, which plays an essential role in the innate immune response to HDM allergens [25]. Airway epithelial cells, expressing TLR4, serve as the first line of defense against HDM allergens [26,27], recognizing and activating downstream signaling cascades that lead to Th2 and Th17 cytokine production. CycloZ treatment effectively decreased TLR4 and downstream modulators and targets, including TRAM, TRIM, IRAKs, NF-κB, and AP-1. This finding supports the idea that CycloZ may interfere with the initial steps of the allergic response, potentially dampening the cascade of inflammation associated with chronic allergic asthma.

While CycloZ effectively downregulated the TLR4 pathway and Gata3-mediated Th2 response, the precise molecular interactions through which CycloZ suppresses these pathways remain to be elucidated. Future mechanistic studies are necessary to understand whether CycloZ directly binds or modifies TLR4 signaling components or acts via other cellular mechanisms to reduce allergic inflammation.

Despite potential long-term side effects such as adrenal suppression [28], FP is still the most commonly used corticosteroid in asthma medications due to its powerful anti-inflammatory effects [29]. However, we found that CycloZ was similarly effective or even better than FP at reducing allergic inflammation and Th2 responses, yet with fewer adverse effects. Long-term corticosteroid use can lead to significant side effects, making CycloZ a particularly attractive alternative due to its favorable safety profile observed in preclinical and clinical studies [22,30,31,32].

There are two main limitations to consider in this study. First, the use of a single allergen (HDMs) limits the generalizability of these findings, as other allergens may activate different immune pathways. Additionally, while the mouse model used mimics certain features of human asthma, it does not fully recapitulate the complex human immune environment. Further studies in other animal models or humanized models would enhance the clinical relevance of these findings. Another limitation is the short duration of the study; while we observed strong anti-inflammatory effects in both acute and chronic models, longer-term studies are needed to assess CycloZ’s potential for chronic asthma management and any delayed adverse effects.

Our findings address key gaps in asthma research and treatment. Current therapies, such as corticosteroids like fluticasone propionate (FP), offer symptom relief but are limited by variable efficacy and risks associated with long-term use, including adrenal suppression. Effective non-steroidal alternatives targeting both inflammation and allergen recognition are lacking. CycloZ bridges this gap by dual mechanisms: it mitigates TLR4-driven immune triggers of allergic asthma and reduces downstream Th2-mediated inflammation, notably decreasing cytokines like IL-4 and IL-13. Unlike FP, which focuses on inflammation, CycloZ’s targeting of the TLR4 pathway addresses the root cause of allergic responses. Preclinical data highlight CycloZ as a potentially safer, more effective alternative to FP, particularly in chronic asthma models, paving the way for transformative advances in asthma treatment.

## 5. Conclusions

In this study, we demonstrated the therapeutic potential of CycloZ in both acute and chronic mouse models of allergic asthma. CycloZ significantly alleviated airway inflammation by reducing inflammatory cell infiltration, suppressing Th2 cytokine production, and downregulating mucus-associated gene expression. Furthermore, CycloZ’s ability to inhibit the TLR4 signaling pathway highlights its dual role in modulating both innate and adaptive immune responses. Compared to FP, a widely used corticosteroid, CycloZ exhibited similar or superior efficacy in reducing asthma-associated inflammation while potentially offering a better safety profile. This positions CycloZ as a promising alternative or adjunct therapy for managing allergic asthma. Despite these findings, there are limitations to our study. The use of HDMs as the sole allergen model and the short experimental timeframe restrict the generalizability of our results. Additionally, the precise molecular mechanisms underlying CycloZ’s action remain to be fully elucidated. Future studies should investigate the long-term effects of CycloZ and its efficacy across various allergen models to enhance the clinical relevance of these findings. Nevertheless, our results provide strong preclinical evidence for the development of CycloZ as a novel therapeutic option for asthma patients.

## Data Availability

The original contributions presented in this study are included in the article/Supplementary Materials. Further inquiries can be directed to the corresponding author.

## Supplementary Materials

The following supporting information can be downloaded at: https://www.mdpi.com/article/10.3390/cells13232034/s1, Figure S1: HDM dose response effects on allergic cytokine expression in male and female mice.; Figure S2: Effect of CycloZ on HDM- and OVA-induced male acute asthma model mice.; Table S1: Gene specific primer sets for Realtime PCR.
