# Supplementary material for: CycloZ Suppresses TLR4-Driven Inflammation to Reduce Asthma-Like Responses in HDM-Exposed Mouse Models
Source: Cells. 2024 Dec 9;13(23):2034. doi: 10.3390/cells13232034 (PMC11640671; doi:10.3390/cells13232034)
Supplement: Supplementary file 1 [file cells-13-02034-s001.zip › cells-3290735-supplementary.pdf]

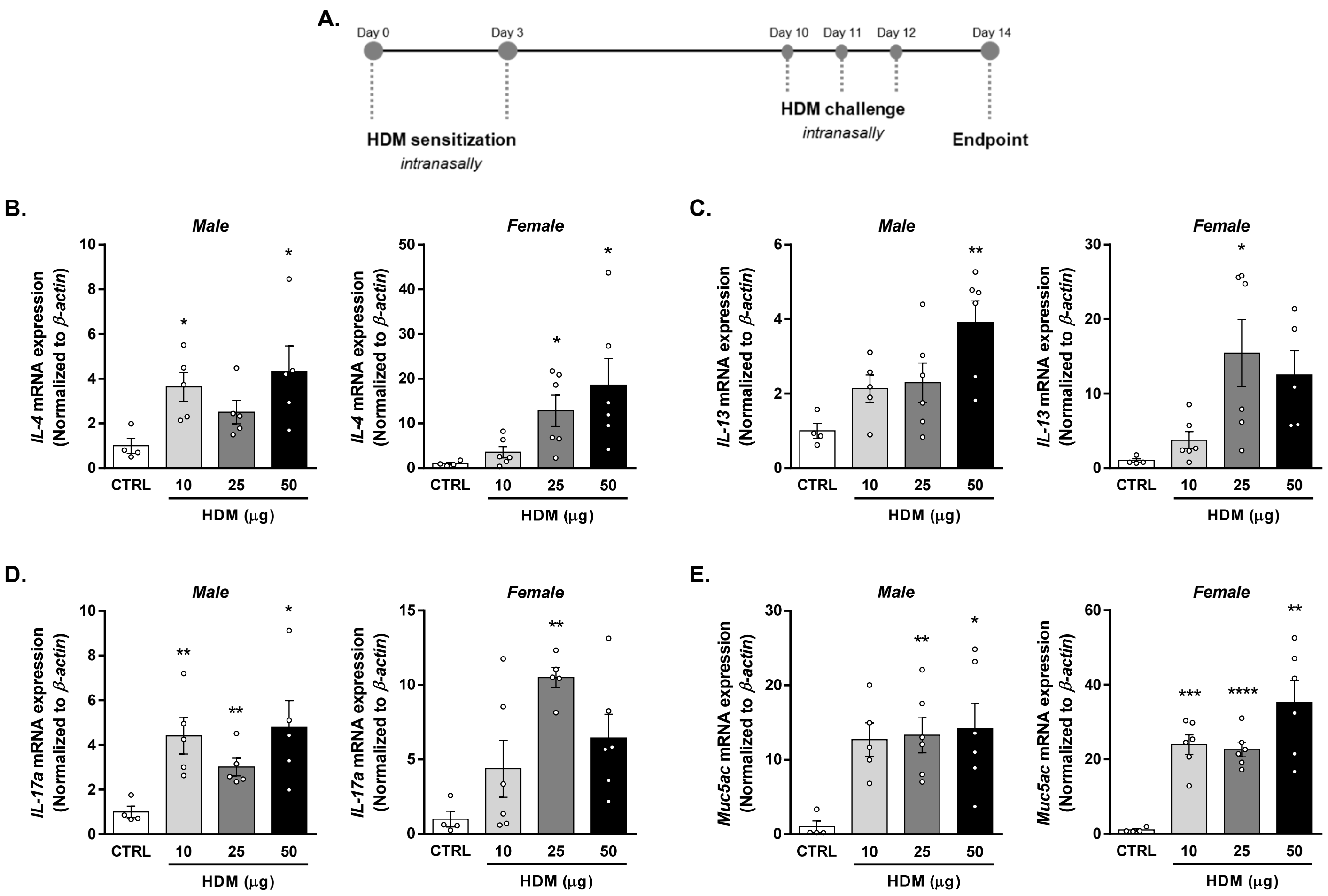

Figure S1. HDM dose response effects on allergic cytokine expression in male and female mice.

7-week-old male and female Balb/c mice were exposed to HDM extract and sacrificed according to the acute model protocol (A). In each sex, groups consisted of CTRL n=4, HDM 10μg n=6, HDM 25μg n=6, HDM 50μg n=6. Lung cytokine expression was analyzed by real-time qPCR. (B) *IL-4*. (C) *IL-13*. (D) *IL-17a*. (E) *Muc5ac*. Data are shown as mean ± SEM. Unpaired Student's t-tests. *p < 0.05, **p < 0.01, ***p < 0.001, ****p < 0.0001 compared to CTRL group.

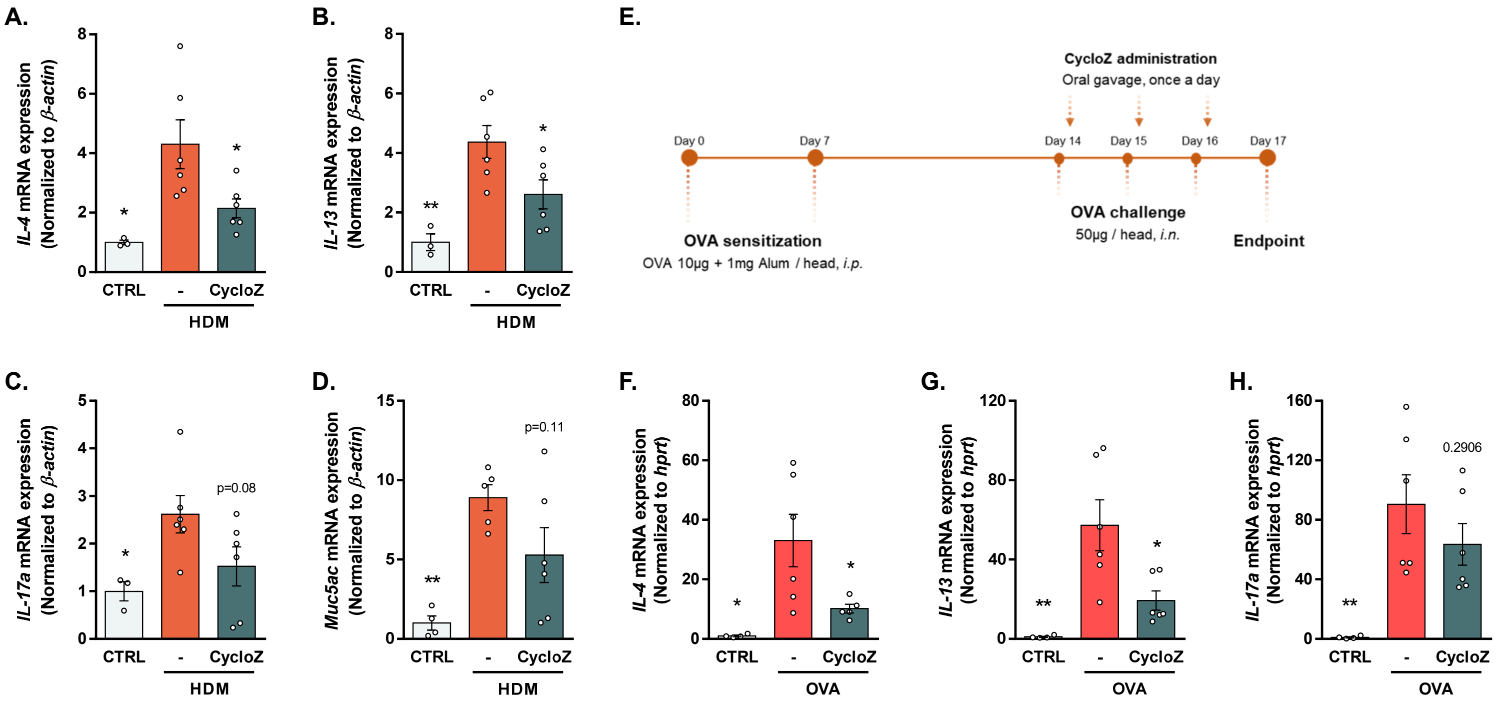

Figure S2. Effect of CycloZ on HDM- and OVA-induced male acute asthma model mice.

(A-D) 7-week-old male Balb/c mice were treated with HDM and CycloZ according to the acute model protocol (Figure 1A). Groups consisted of CTRL n=3, HDM+Vehicle n=6, HDM+CycloZ n=6. Lung cytokine expression was analyzed by real-time qPCR. (A) *IL-4*. (B) *IL-13*. (C) *IL-17a*. (D) *Muc5ac*. (E-H) 8-week-old male C57BL6/J mice were used for OVA-induced acute asthma model. Groups consisted of CTRL n=4, OVA+Vehicle n=6, OVA+CycloZ n=6. (E) Experimental design for OVA-induced acute asthma model. (F-H) mRNA expression of lung *IL-4, IL-13*, and *IL-17a*. Data are shown as mean ± SEM. Unpaired Student's t-tests. *p < 0.05, **p < 0.01 compared to HDM+Vehicle or OVA+Vehicle group.

Table S1. Gene specific primer sets for Realtime PCR

Gene	Forward (5' to 3')	Reverse (5' to 3')
IL-4	TCGGCATTTTGAACGAGGTC	CGTTGCTGTGAGGACGTTTG
IL-5	TCAAACTGTCCGTGGGGGTA	CCACACTTCTCTTTTTGGCGG
IL-13	CCATCTACAGGACCCAGAGGA	TTTTGGTATCGGGGAGGCTG
IL-17a	CCTCACACGAGGCACAAGT	TGAAGCTCTCCCTGGACTCA
IL-33	ATGGGAAGAAGCTGATGGTG	CCGAGGACTTTTTGTGAAGG
Muc5ac	TGTGCCTGCCTGTACAATGGG	CATGTGTTGGTGCAGTCAGTAGA
Gata3	ACAGAAGGCAGGGAGTGTGT	GTCTGACAGTTGGCACAGGA
Stat6	CTCTGTGGGGCCTAATTTCCA	GCATCTGAACCGACCAGGAAC
NF-ATc1	GGTGCCTTTTGCGAGCAGTATC	CGTATGGACCAGAATGTGACGG
c-maf	AGGAGGTGATCCGACTGAAGCA	TCTCCTGCTTGAGGTGGTCTAC
TLR4	CATCCAGGAAGGCTTCCACA	GGCGATACAATTCCACCTGC
TRAM	CGCCTGCCAAGCTAGAACT	AGGTTGCCTGGGACATTACA
TRIM	CTGTGGAACAAGGAACAGCAG	TGTGCCTCCCAATGAAATCCT
IRAK1	CTGGATGAGCCTCAGATCCC	ACTTTCCACTGGCTGGTTGG
IRAK4	ACAGCGACAACCTGTGCTTA	GAGCAACCTTGCACCTTGTG
NF-κB p105	GAGAAGAACAAGAAATCCTACCCAC	TCCATTTGTGACCAACTGAACG
IRF3	TGGGCAGCACAGCT ACATGA	GCCCATTGCCCAGCCCTT
c-Jun	CGCTCGGCTAGAGGAAAAAG	TGTTCCCTGAGCATGTTGG
c-fos	GAGCCAGTCAAGAGCATCAG	GCCTAGATGATGCCGGAAA
β-actin	GGGAAGGTGACAGCATTG	ATGAAGTATTAAGGCGGAAGATT
hprt	AAATGTCAGTTGCTGCGTCC	TCTACCAGAGGGTAGGCTGG
